# Xanthan Gum Capped ZnO Microstars as a Promising Dietary Zinc Supplementation

**DOI:** 10.3390/foods8030088

**Published:** 2019-03-02

**Authors:** Alireza Ebrahiminezhad, Fatemeh Moeeni, Seyedeh-Masoumeh Taghizadeh, Mostafa Seifan, Christine Bautista, Donya Novin, Younes Ghasemi, Aydin Berenjian

**Affiliations:** 1 Department of Medical Nanotechnology, School of Advanced Medical Sciences and Technologies, Shiraz University of Medical Sciences, Shiraz 71348, Iran; a_ebrahimi@sums.ac.ir; 2 Department of Pharmaceutical Biotechnology, School of Pharmacy and Pharmaceutical Sciences Research Center, Shiraz University of Medical Sciences, Shiraz 71348, Iran; a.ebrahiminezhad@yahoo.com (F.M.); Taghizadehs@sums.ac.ir (S.-M.T.); 3 School of Engineering, Faculty of Sciences and Engineering, University of Waikato, Hamilton 3216, New Zealand; mostafa.seifan@waikato.ac.nz (M.S.); christinebautista0621@yahoo.com (C.B.); donya.dnov@gmail.com (D.N.)

**Keywords:** xanthan gum, ZnO microparticles, ZnO microstars, bacteria, supplementation, food fortification

## Abstract

Zinc is one of the essential trace elements, and plays an important role in human health. Severe zinc deficiency can negatively affect organs such as the epidermal, immune, central nervous, gastrointestinal, skeletal, and reproductive systems. In this study, we offered a novel biocompatible xanthan gum capped zinc oxide (ZnO) microstar as a potential dietary zinc supplementation for food fortification. Xanthan gum (XG) is a commercially important extracellular polysaccharide that is widely used in diverse fields such as the food, cosmetic, and pharmaceutical industries, due to its nontoxic and biocompatible properties. In this work, for the first time, we reported a green procedure for the synthesis of ZnO microstars using XG, as the stabilizing agent, without using any synthetic or toxic reagent. X-ray diffraction (XRD), Fourier transform infrared spectroscopy (FTIR), and transmission electron microscopy (TEM) were used to study the structure, morphology, and size of the synthesized ZnO structures. The results showed that the synthesized structures were both hexagonal phase and starlike, with an average particle size of 358 nm. The effect of different dosages of XG-capped ZnO nanoparticles (1–9 mM) against Gram-negative (*Escherichia coli*) and Gram-positive (*Bacillus licheniformis*, *Bacillus subtilis*, and *Bacillus sphaericus*) bacteria were also investigated. Based on the results, the fabricated XG-capped ZnO microstars showed a high level of biocompatibility with no antimicrobial effect against the tested microorganisms. The data suggested the potential of newly produced ZnO microstructures for a range of applications in dietary supplementation and food fortification.

## 1. Introduction

Zinc plays many essential roles in all life forms [1], and is part of the major subgroup of micronutrients that are beneficial in human nutrition and health [2]. In the human diet, the main sources of zinc are animal products [3], rice, wheat, and soybean [4]. However, there has been an insufficient dietary supply of the element, which has become the primary cause of zinc deficiency. Studies have revealed that the concentrations of zinc mineral elements in edible parts have decreased over the last 50 years [5], and that high levels of dietary inhibitors may suppress the absorption of zinc [6]. Zinc deficiency has an effect on organ systems such as the epidermal, gastrointestinal, central nervous, immune, skeletal, and reproductive systems [7]. The literature has also determined that zinc deficiency can impair physical growth, and zinc-dependent metabolic functions [2].

It is essential for an individual to consume a certain level of zinc, as the zinc deficiency can have several consequences on human health. There are currently four main approaches to increasing the intake of zinc in the human diet: (1) direct supplementation, (2) food fortification, (3) dietary diversification, and (4) crop biofortification [5]. To compensate for insufficient zinc intake, the major suggested route is through the intake of oral zinc supplementation. The forms of zinc that are frequently used in supplements are zinc sulfate, zinc oxide, zinc acetate, and zinc gluconate [8]. However, zinc supplements are not well tolerated by all patients, even in high doses, due to inadequate intestinal zinc absorption [6]. Currently, there is great attention on the use of micro and nanoparticles to fortify micronutrients, due to their ultra-small size which aids intestinal absorption. Micronization is another effective technique to increase the digestive absorption of poorly soluble nutrients and drugs. It has been reported that the decrease in the particle size, up to a few microns, can substantially enhance the rate of dissolution and can consequently improve the bioavailability and clinical efficacy [9]. Although, in contrast to metal salts, which are commonly prescribed, metal and metal oxide particles are more tolerable for patients, and therefore, higher dosages can be used [6]. 

Nanometer-sized particles of zinc oxide (ZnO) have many applications in various areas due to their unique and superior chemical and physical properties compared to bulk ZnO. Over the last decade, massive investigations have also been performed to evaluate the effect of ZnO nanoparticles on the growth of microbial communities. For example, Ramesh et al. [10] investigated the antibacterial activity of green-synthesized ZnO nanoparticles with an average diameter of 29.79 nm. The zone inhibition method was used to explore the antibacterial activity of ZnO nanoparticles against Gram-positive (*Staphyloccus aureus*) strains, and Gram-negative (*Salmonella paratyphi*, *Vibrio cholerae*, *Escherichia coli*) strains. Authors have concluded that the ZnO nanoparticles exhibit good antibacterial activities by decreasing the cell growth. Banoee et al. [11] conducted a similar study which explored the antibacterial activity of ZnO nanoparticles with an average size of 20–45 nm against *S. aureus* and *E. coli*. It was observed that the presence of fabricated ZnO nanoparticles enhanced the antibacterial activity of ciprofloxacin, and increased the inhibition zone by 27% and 22%, for *S. aureus* and *E. coli*, respectively. However, the availability of 500 µg/disk ZnO nanoparticles decreased the antibacterial activity of amoxicillin, penicillin G, and nitrofurantoin in *S. aureus*. The antibacterial behavior of *Vitex negundo* extract assisted ZnO nanoparticles against *S. aureus* (ATCC 11632) and *E. coli* (ATCC 10536), and was studied by Ambika et al. [12] using the agar well diffusion method. The results showed that synthesized ZnO nanoparticles have high antibacterial activity. It was shown that the availability of ZnO nanoparticles contributed to a zone of inhibition of 19 mm and 16 mm for *S. aureus* and *E. coli*, respectively.

To be considered as a supplement or to be used for food fortification, the fabricated ZnO must have no negative effect on gastrointestinal microbiota. Intestinal bacteria play a key role in both digestion and regulation of the immune system. For example, they are responsible for maintaining immune and metabolic homeostasis, and protecting against pathogens [13]. There are different chemical, nutritional, and immunological factors that can affect the population of human microbiota. In this regard, the properties of metallic elements such as ZnO can significantly limit gastrointestinal bacterial growth. Moreover, it is obvious that antimicrobial properties of the metallic particles can be varied by changing their physicochemical properties. In this context, the shape, particle size, and capping material are the most effective parameters [14,15]. 

To date, there are no reports on the successful fabrication of biocompatible ZnO particles for application as a dietary supplement. Therefore, this study was performed to synthesize a biocompatible ZnO particle using a natural heteropolysaccharide, xanthan gum (XG), as a stabilizer, thickener, and emulsifier without using any toxic chemicals. In contrast to the currently available zinc supplements, the fabrication of biocompatible micro-sized ZnO particles may be a promising form of zinc supplementation, as the reduction of metal elements to a few hundreds of microns, can substantially enhance their intestinal absorption.

## 2. Materials and Methods

### 2.1. Chemicals

The zinc acetate dehydrate was obtained from Merck Millipore (Darmstadt, Germany). The Ammonium hydroxide, XG and LB broths (Lysogeny broth) were purchased from Sigma-Aldrich (St. Louis, Missouri, USA). All the solutions used for the fabrication of nanoparticles were prepared using ultrapure water through a Millipore water purification system (Milli-Q, Milford, MA, USA). Ampicillin discs (10 µg) and blank paper discs (6 mm diameter) were obtained from Fort Richard Laboratories (Auckland, New Zealand).

### 2.2. General Procedure for the Synthesis of XG-Capped ZnO Microstars

**Figure 1:**
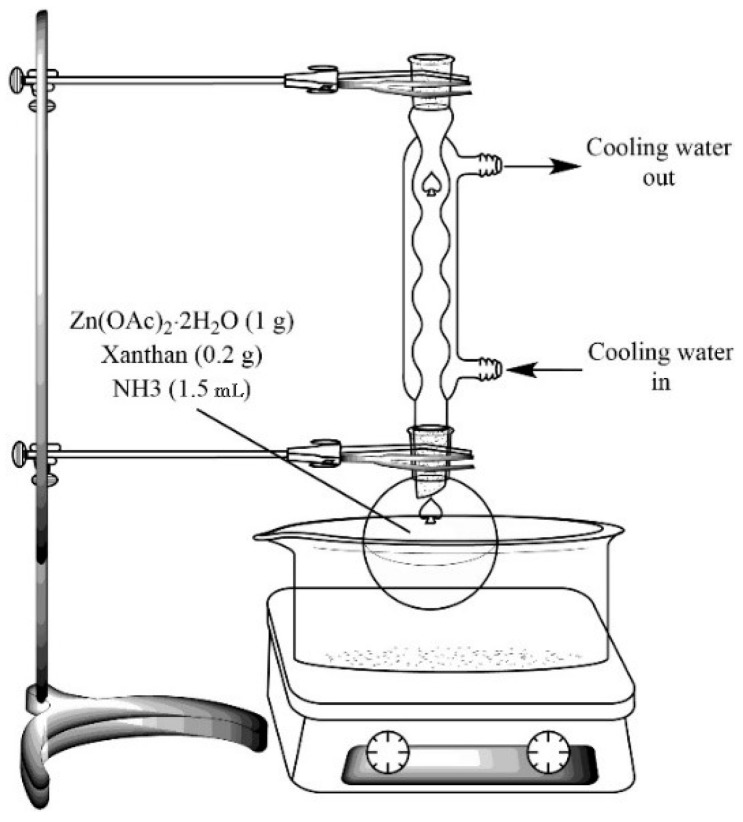
Schematic illustration of the steps involved in the synthesis of xanthan gum (XG)-capped zinc oxide (ZnO) microstars using an efficient hydrothermal method.

The concentrations of Zn^2+^, XG, and NH_4_OH, along with the reaction time and temperature, were chosen based on preliminary studies as they affected the size and shape of the resulting particles. In the first step, Zn(OAc)_2_·2H_2_O (as Zn^2+^ source, 1 g) and XG (0.2 g) were dissolved in 140 mL ultrapure water at room temperature. Then 1.5 mL ammonium hydroxide (NH_4_OH, 25%) was added dropwise to the solution. As schematically shown in Figure 1, the mixture was refluxed for 6 h at 80 °C, and cooled to stop the reaction. After that, the product was centrifuged and washed with deionized water. Then, 90 mL of ultrapure water was added and the mixture was refluxed for 9 h. The resulting material was washed with deionized water and dried in an oven at 60 °C for 24 h. 

### 2.3. Material Characterization

Different analytical techniques were used to visualize and characterize the synthesized ZnO microstars. In order to analyze the size and morphology of the fabricated microstars, transmission electron microscopy (TEM) (Philips, Eindhoven, The Netherlands, CM10, HT 100 KV) was used. The verification of the XG-capped ZnO particles was performed using Fourier transform infrared (FTIR) spectroscopy (PerkinElmer Spectrum One, Waltham, Massachusetts, USA). The analysis was carried out using KBr pellets, and the data were collected for a range of 4000–400 cm^−1^. X-ray diffraction (XRD) analysis was performed to characterize the synthesized microstars using a Siemens D5000 X-ray powder diffractometer. The data acquisition was carried out for an exploration range (2θ degree) of 10–80°.

### 2.4. Microorganism and Bacterial Culture Conditions

The following bacteria were revived according to the procedure provided by the New Zealand culture collection: *Escherichia coli* (NZRM 250), *Bacillus licheniformis* (ATCC 9789), *Bacillus subtilis* (ATCC 9799) and *Bacillus sphaericus* (ATCC 4525). All freeze-dried bacteria were revived in Luria-Bertani medium by incubating the culture overnight at 37 °C and 200 rpm.

### 2.5. Determination of Antimicrobial Activity of XG-Capped ZnO Microstars

Prepared ZnO microstars were suspended in distilled water and constantly treated with an ultra sonicator (Qsonica-Q800R, Qsonica, Newtown, Connecticut, USA), in an ultrasonic amplitude of 75% (500 W), until a uniform colloidal suspension was formed, which yielded a 9 mM stock solution. Serial dilution was then carried out, which resulted in 1, 3, 6, and 9 mM of freshly prepared XG-capped ZnO particle suspensions. The antimicrobial activities of the suspensions were investigated by studying the inhibition zones (agar disk-diffusion) on a comparative basis, against both Gram-negative and Gram-positive bacteria [16]. Briefly, 400 µL of each grown bacteria was spread over the entire agar plate surface. Then, the blank filter paper disks were soaked in each ZnO suspension and placed in the center of the plate. A blank disc, and an ampicillin disc (10 µg), with a diameter of 6 mm, was used as a negative and positive control, respectively. The plates were incubated at 37 °C for 24 h. The plates were examined for evidence of zones of inhibition and the diameter was measured using a millimeter ruler.

## 3. Results and Discussion

### 3.1. Synthesis of ZnO Microstars

The zinc oxide nanoparticle is an inorganic compound widely used in various applications, and can be synthesized through different methods, such as mechanochemical, controlled precipitation, precipitation in the presence of surfactants, emulsion, microemulsion, sol-gel, microwave assisted, solvothermal, and hydrothermal processes [17]. In this study, we used the hydrothermal method to fabricate XG-capped ZnO microstars, as it offered a straightforward and environmentally friendly technique without additional processing of the product such as grinding or calcination. The particle growth process can be expressed according to the following reactions:

(1) $$Zn{\left(CH_{3}COO\right)}_{2}\cdot 2H_{2}O\to 2CH_{3}COO^{-}+Zn^{2+}+2H_{2}O$$

(2) $$CH_{3}COO^{-}+\;H_{2}O\to CH_{3}COOH+OH^{-}$$

(3) $$NH_{3}+\;H_{2}O↔NH_{4}^{+}+OH^{-}$$

(4) $$Zn^{2+}+4NH_{4}^{+}\to {\left[Zn{\left(NH_{3}\right)}_{4}\right]}^{2+}$$

(5) $$Zn^{2+}+2OH^{-}\to Zn{\left(OH\right)}_{2}$$

(6) $$Zn^{2+}+4OH^{-}\to Zn{\left(OH\right)}_{4}^{2-}$$

(7) $$Zn{\left(OH\right)}_{2}+2OH^{-}\to Zn{\left(OH\right)}_{4}^{2-}$$

(8) $$Zn{\left(OH\right)}_{4}^{2-}\to ZnO+2OH^{-}+H_{2}O$$

(9) $${\left[Zn{\left(NH_{3}\right)}_{4}\right]}^{2+}+2OH^{-}\to ZnO+4NH_{3}+H_{2}O$$

In the first step, the zinc acetate reacted with ultrapure water and the corresponding zinc and acetate ions were formed as shown in reaction (1). The acetate ions underwent hydrolysis with water and released the hydroxide anions as shown in reaction (2). When ammonia solution was added, it formed ammonium and hydroxide ions, as shown in reaction (3) [18]. During the process, the zinc ions reacted with ammonium and hydroxide ions. Tetraamine zincate, zinc hydroxide, or tetrahydroxozincate were formed according to reactions (4–6). Due to dehydration of the complex in the alkaline medium, ZnO nuclei are crystallized, as a result of the reaction of zinc hydroxide complex ions and tetraamine zincate complex ions, with hydroxide ions, as shown in reactions (8 and 9) [19].

### 3.2. Characterization of ZnO Microstars

#### 3.2.1. FTIR Spectra Analysis

**Figure 2:**
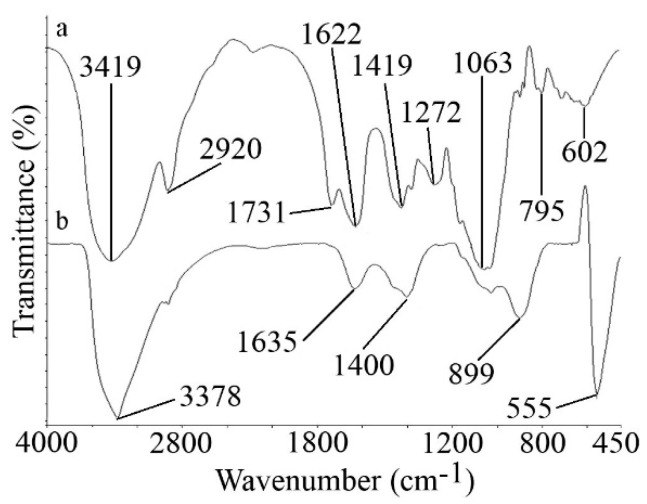
Fourier transform infrared spectroscopy (FTIR) spectra of (**a**) XG and (**b**) XG-capped ZnO microstars.

The FTIR spectrum of the XG and ZnO microstars in the region of 450–4000 cm^−1^ is shown in Figure 2. The major absorption bands present in the FTIR spectrum of pure XG were shown in Figure 2a. The broad absorption peak at 3419 cm^−1^ was due to the stretching vibrations of O–H groups. The band of 2920 cm^−1^ could be assigned to the absorption of symmetrical and asymmetrical stretching of methyl and methylene groups in XG. The peaks at 1731 and 1622 cm^−1^ could be due to the characteristic asymmetrical stretch of the carboxylate group and the carbonyl group, respectively. The peak at 1419 cm^−1^ was assigned to the absorption of symmetrical stretching of the carboxyl group. Figure 2b shows the FTIR spectra of the XG-capped ZnO microstars. The band located at 555 cm^−1^ correlated to the stretching mode of ZnO. In the presence of ZnO microstars the band at 3419 cm^−1^ shifted to 3378 cm^−1^, indicating the interaction of Zn with the OH group of XG. The interactions among the resultant ZnO nanoparticles, and oxygen atoms of hydroxyl, carboxyl, and carbonyl group led to corresponding changes in the positions, and in the strengths of spectrum of the XG.

#### 3.2.2. XRD Analysis

**Figure 3:**
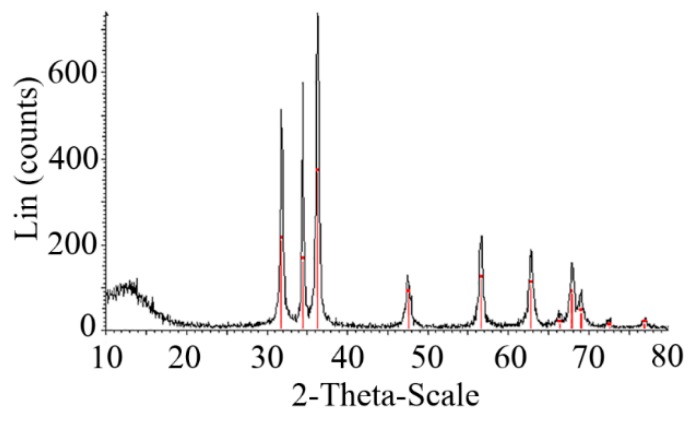
X-ray diffraction (XRD) spectra of XG-capped ZnO microstars.

To confirm the purity and to determine the crystal structure of the particles, X-ray diffractometry was used. As can be concluded from Figure 3, all the diffraction peaks are well indexed as hexagonal wurtzite-structures (JCPDS card No. 36–1451), and no characteristic peaks were observed other than ZnO. The strong and narrow diffraction peaks illustrated that the nanoparticles enjoyed high crystallinity and purity. 

#### 3.2.3. TEM Analysis

**Figure 4:**
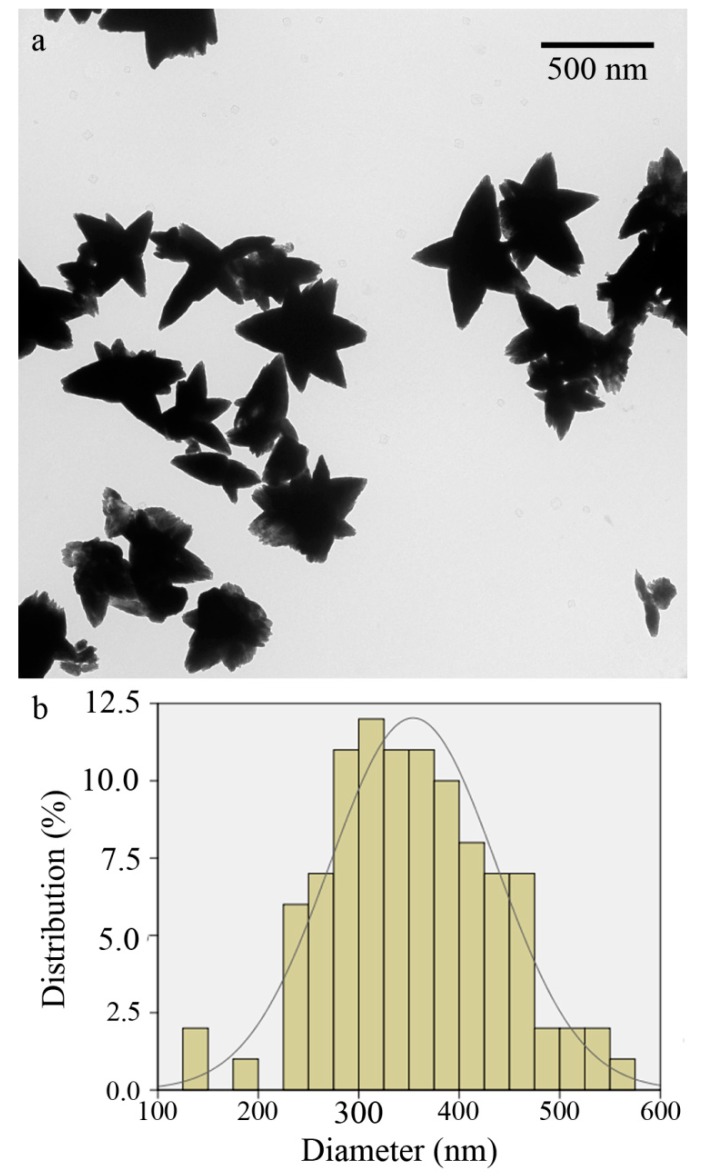
(**a**) Transmission electron microscopy (TEM) micrograph of XG-capped ZnO microstars and (**b**) corresponding histogram with normal distribution.

The size, distribution, shape, and morphology of XG-capped ZnO microstars were evaluated by TEM (Figure 4a). The micrograph was analyzed using the ImageJ software version 1.47v, an image analysis software developed by the NIH (http://imagejnihgov/ij/).The particle size histogram, with normal distribution is provided in Figure 4b. The prepared particles were starlike microstructures, ranging from 140–553 nm in diameter, with a mean particle size of 358 nm. 

Shape and size of the prepared particles can be dictated by the presence of XG in the synthesis reaction. Using a similar method, Hosseini-Sarvari et al. [20] obtained ZnO nano-rods from Zn(OAc)_2_·2H_2_O and NH_4_OH (25%), in the presence of the water-soluble linear polymer (PEG 2000), instead of XG. The presence of PEG 2000 was found to affect both the shape and the size of the resulting ZnO particles. It was reported that the availability of surfactant PEG 2000 contributed to the formation of hexagonal wurtize-structured nanorod particles with a crystallite size of 17–20 nm.

### 3.3. Microbicidal Activity of Fabricated XG-Capped ZnO Microstars

Although ZnO nanoparticles have great potential as a competitive zinc supplement, previous studies showed that the ZnO nanoparticles have an adverse effect on the growth and metabolism of different microorganisms. This antimicrobial characteristic significantly decreased the effectiveness, and functionality of ZnO nanoparticles, as they inhibited the gastrointestinal microbiota.

**Figure 5:**
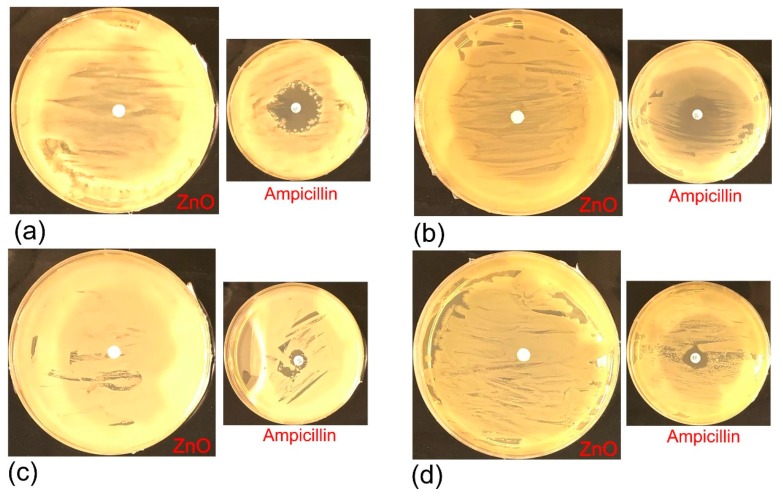
XG-capped ZnO microparticles and Ampicillin against (**a**) *Bacillus subtilis* (**b**) *Bacillus sphaericus* (**c**) *Bacillus licheniformis* and (**d**) *Escherichia coli.*

Surface coating with biocompatible XG and increasing the particle size to micro scales may offer a better solution to address the negative effect of ZnO particles on the microorganisms. Therefore, the antibacterial activity of the XG-capped ZnO microparticles against both Gram-positive and Gram-negative bacteria were further investigated using the agar diffusion method. As shown in Figure 5, the presence of Ampicillin as a control resulted in an inhibition zone in all the strains. Among the investigated bacteria, *B. lcheniformis* showed the least inhibition zone (11 mm). The largest inhibition zone was observed for *B. subtilis* (32.5 mm), followed by *B. sphaericus* (24 mm) and *E. coli* (14.5 mm). However, the agar plates for the different concentrations of XG-capped ZnO microstars did not show any inhibition zones (Figure 5), which implied that the synthesized particles had no negative effect on the bacterial growth and metabolism.

Previous studies have reported high antibacterial activities for naked ZnO nanoparticles against both Gram-positive and Gram-negative bacteria [21]. For instance, it was reported that the growth inhibition of bacteria increased with an increase in naked ZnO nanoparticle concentrations [22]. It was noted that the size of the inhibition zone was dependent upon the type of bacteria, the size, and the concentrations of ZnO nanoparticles [23]. Although the exact mechanism of ZnO antibacterial activity is still not clear, the literature has determined hypothetical mechanisms that could explain this phenomena. One of the hypothetical mechanisms is the formation of reactive oxygen species (ROS). Studies have shown that aquatic ZnO nanoparticles produced an augmented level of ROS, which has been reported as the major cause of nontoxicity. This increased level was due to the generation of hydrogen peroxide (H_2_O_2_) molecules, which were capable of entering the membrane, where they could damage or kill the bacteria [24]. Another proposed mechanism was the release of zinc ions (Zn^2+^) and their adhesion to the cell membrane, which caused mechanical damage to the cell wall. A further suggestion of the mechanism of ZnO antibacterial activity was the disruption of the membrane, resulting in membrane dysfunction and the internalization of ZnO nanoparticles into the bacteria [25].

In the present study we reported the successful fabrication of XG-capped ZnO microstars with no antibacterial activity. This behavior could be justified by an increase in the size of the prepared particles from the nano to the micro scale. It was obvious that the antimicrobial properties of the metallic particles were severely influenced by the particle size and shape [14,15]. Increases in the particle size usually resulted in a decrease of the antimicrobial properties. However, the antimicrobial properties of metallic particles were not only under the control of particle size, and particle size was not the dominant parameter. Attention should be paid to the other physical and chemical properties of the particles [15]. Surface capping, and using a biocompatible material, could minimize the inhibitory effects of metallic particles on bacterial growth. XG is a particular exopolysaccharide secreted by the fermentation of the bacterium *Xanthomonas campestris* in aerobic conditions, from sugar cane, corn or their derivatives [26]. It is a natural heteropolysaccharide, with branched chains and acidic characteristics, consisting of repeated pentasaccharide units with two glucose units, two mannose units, and one glucuronic acid unit in the ratio of 2.8:2.0:2.0 [27].

There are two proposed mechanisms for the stabilization of inorganic particles using natural gums. The first mechanism was the result of XG adsorbed through the surface of particles, which caused steric repulsion among the particles. The second mechanism involved XG increasing the viscosity of the particle suspensions, hence gradually decreasing the aggregation processes [28,29]. XG was highly soluble in both hot and cold water, hydrated quickly, and was stable over a wide range of temperature, acidic, and alkaline conditions [30,31]. XG has a wide range of applications in the food industry due to its nontoxic and biocompatible properties, thickening properties, and high suspending ability [31,32,33,34]. Therefore, the XG-capped ZnO microstars that were synthesized in this study may be a possible approach in creating a dietary zinc supplement that could facilitate zinc intake in the future.

## 4. Conclusions

A green and -efficient method for the synthesis of XG-capped ZnO microstars was developed using XG as the stabilizing agent. TEM, XRD, and FTIR were used to characterize the fabricated nanoparticles. The antibacterial activity of the fabricated XG-capped ZnO microstars were also investigated using the agar diffusion method. It was observed that all tested strains were susceptible to Ampicillin as a control. However, different concentrations of XG-capped ZnO microstars did not show any inhibitory effects against both Gram-positive and Gram-negative bacteria. This behavior could be justified by the particles at the micro scale, and the presence of biocompatible XG capping on ZnO microstars.
